# Preparation, Structural Features and *in vitro* Immunostimulatory Activity of a Glucomannan From Fresh *Dendrobium catenatum* Stems

**DOI:** 10.3389/fnut.2021.823803

**Published:** 2022-02-01

**Authors:** Jingjing Liu, Luyao Yu, Chun Wang, Yuefan Zhang, Hangxian Xi, Jinping Si, Lei Zhang, Jingkun Yan

**Affiliations:** ^1^State Key Laboratory of Subtropical Silviculture, Zhejiang A&F University, Hangzhou, China; ^2^Department of Pharmaceutical Botany, School of Pharmacy, Second Military Medical University, Shanghai, China; ^3^Key Laboratory of Healthy Food Development and Nutrition Regulation of China National Light Industry, School of Chemical Engineering and Energy Technology, Dongguan University of Technology, Dongguan, China; ^4^Biomedical Innovation R&D Center, School of Medicine, Shanghai University, Shanghai, China

**Keywords:** *Dendrobium catenatum*, polysaccharide, three-phase partitioning, structure, rheological property, immunostimulatory activity

## Abstract

*Dendrobium catenatum* polysaccharides (DCPs) have attracted attention due to their multiple physiological activities and health benefits. In this study, a novel water-soluble DCP was obtained from fresh *D. catenatum* stems through three-phase partitioning and ethanol precipitation at room temperature. Its structural characteristics, rheological property, and *in vitro* immunostimulatory activity were evaluated. Results demonstrated that DCP was a homogenous polysaccharide with a carbohydrate content of 92.75% and a weight-average molecular weight of 2.21 × 10^5^ Da. This polysaccharide is an *O*-acetylated glucomannan comprised by glucose, mannose, and galacturonic acid in a molar ratio of 30.2:69.5:0.3 and mainly comprises (1→4)-β-D-mannopyranosyl (Manp), 2-*O*-acetyl-(1→4)-β-D-Manp, (1→6)-α-D-glucopyranosyl (Glcp), and (1→4)-α-D-Glcp residues. DCP exhibits an extended rigid chain in an aqueous solution and favorable steady shear fluid and dynamic viscoelastic behaviors. *In vitro* immunostimulating assays indicated that DCP activates RAW264.7 cells, thus markedly promoting macrophage proliferation and phagocytosis and increasing the levels of nitric oxide, interferon-γ, interleukin-6, and interleukin-1β. Moreover, the presence of O-acetyl group and high *M*_*w*_ in DCP might be responsible for its potent immunostimulatory activity *in vitro*. Therefore, our data suggested that DCP could be developed as a promising immunostimulant in functional food and pharmaceutical industries.

## Introduction

*Dendrobium catenatum* Lindley (*D. catenatum*), generally called Tie-Pi-Shi-Hu in Chinese, is a perennial epiphytic herb belonging to *Orchidaceae* family (1). As a valuable traditional Chinese medicine, *D. catenatum* is commonly consumed as a tonic and a precious food therapy for several thousand years in China according to the ancient Chinese medical book “Shennon*G*′s Materia Medica” (2). This herb has been named as “the gold in medicine” and “the life-saving fairy grass” by the people because it benefits the stomach, nourishes Yin, clears heat, moistens the lungs, and relieves cough (3). Modern pharmacological studies showed that *D. catenatum* exhibits immuno-strengthening, anti-fatigue, antioxidant, promoting digestion, hypoglycemic, and antihypertensive effects (4). These multiple physiological activities and health benefits are attributed to its many bioactive components, including polysaccharides, amino acids, sesquiterpenoids, and alkaloids. As a high quality dietary food, *D. catenatum* has also been extensively applied in porridges, soups, and vegetables and is officially authorized by the China Food and Drug Administration (5).

*D. catenatum* polysaccharides (DCPs) are one of the most important bioactive compounds in *D. catenatum* and exhibit a wide spectrum of biological activities, such as immunostimulation (6), anti-tumor (7), antioxidation (8), anti-diabetes (9), anti-inflammation (10), hepatoprotection (11), cardioprotection (12), and beneficial effects on intestinal microbiota (10, 13). Over the last 2 decades, many DCPs with diverse structures and biological activities have been extracted and isolated from the stems, leaves, and flowers of *D. catenatum* by using different extraction techniques (14–16). Most of these DCPs are classified as 1,4-linked glucomannan or mannoglucan, and their β-configurations are acetylated to varying degrees and locations, with or without branching. *D. catenatum* plant must be dried prior to DCP extraction, but this step increases equipment input and maintenance costs and may affect the physiological activities of DCPs. Existing extraction and separation protocols are often associated with disadvantages of high temperature, laborious work, and low efficiency. Three-phase partitioning (TPP), which typically involves *t*-butanol and (NH_4_)_2_SO_4_, has been extensively applied for the extraction and separation of active ingredients including proteins, enzymes, lipids, polysaccharides, and small-molecular organic compounds (17). In our research, bioactive polysaccharides have been extracted and isolated from fresh okra pods and garlic bulbs by using TPP coupled with non-solvent gradient precipitation methods (18, 19). To our knowledge, no or only a few investigations have focused on the production, structural features, and biological activities of DCPs extracted from fresh *D. catenatum* stems through TPP.

In this work, a novel DCP was extracted and separated from fresh *D. catenatum* stems *via* TPP coupled with ethanol precipitation. Its physiochemical, structural, and rheological characteristics were investigated. *In vitro* immunostimulating activity on RAW264.7 macrophage cells of this DCP was also evaluated. This research provides a theoretical reference for the efficient extraction of DCPs with high purity and bioactivity from fresh *D. catenatum* stems.

## Experimental

### Materials and Chemicals

*D. catenatum* was harvested from the standard greenhouse located in Lin'an District, Hangzhou, Zhejiang province, China (1). Its absolute location is east longitude, 119°26′11″; north latitude, 30°20′30″; altitude, 280 m. The original *D. catenatum* plant was cultivated in a substrate of pine barks, and the stem with moisture content of 76.15% was collected in December 2019. The botanical origin of plant was identified by Professor Jinpin Si from Zhejiang A&F University (Hangzhou, China).

Trifluoroacetic acid (TFA), bovine serum albumin (BSA), deuterium oxide (D_2_O, 99.9%), lipopolysaccharides (LPS), sulfanilamide, and N-(1-napthyl) ethylenodiamine hydrochloride were supplied by Sigma-Aldrich (St. Louis, MO, USA). Cytokine detecting ELISA kits (IL-1β, IL-6 and IFN- γ) were provided by BD Biosciences (Franklin Lakes, NJ, USA). Fluorescein-5-isothiocyanate (FITC)-labeled *Escherichia coli* was procured from Gibco (Invitrogen Corporation, Grand Island, NY, USA).

### Extraction and Separation of DCP

**Figure 1:**
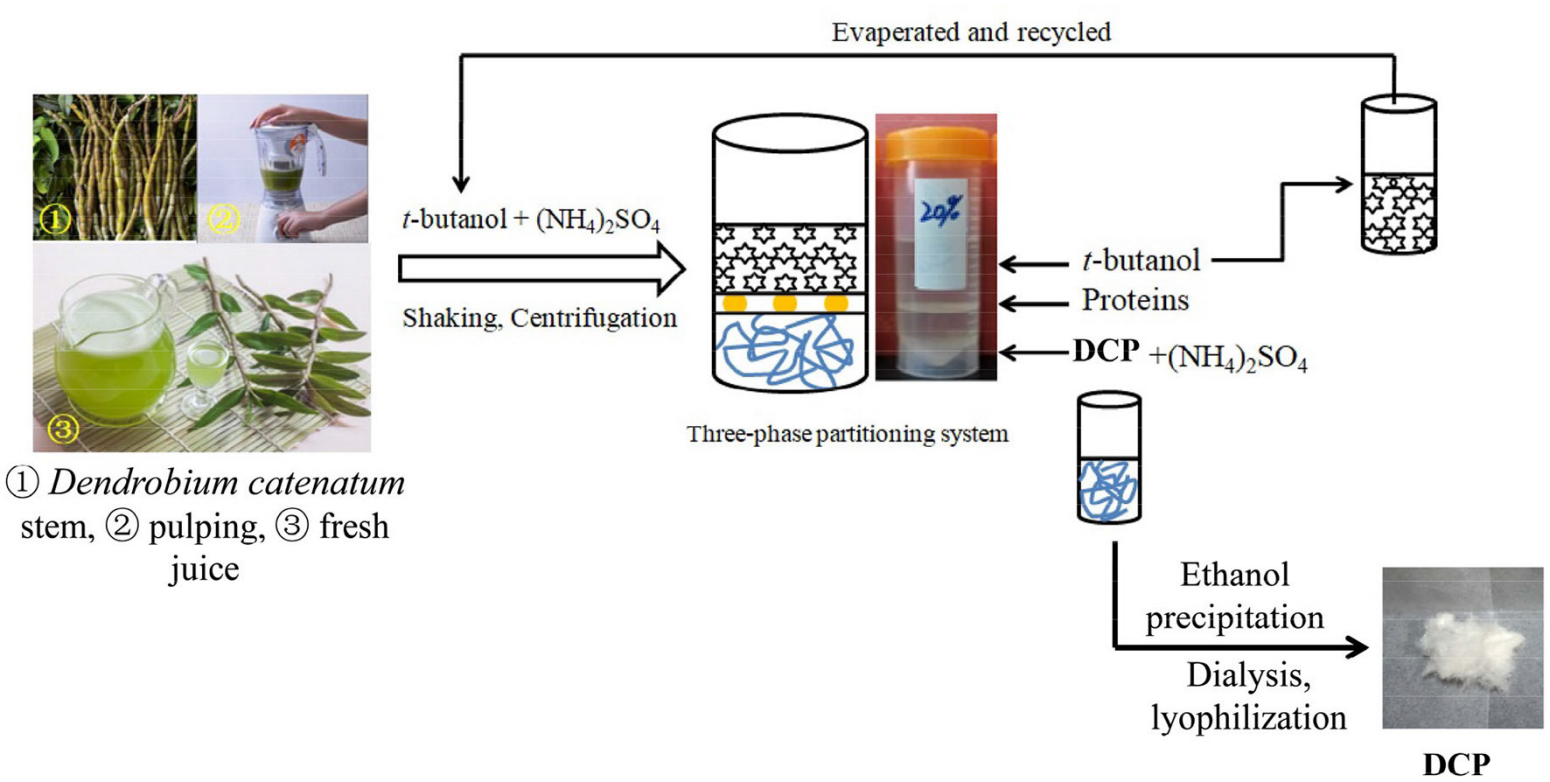
Extraction and isolation procedures for the DCP from fresh *D. catenatum* stems by using TPP and ethanol precipitation.

The extraction and separation of DCP from fresh *D. catenatum* stem were carried out by TPP and ethanol precipitation method according to a previous study (19) with slight modifications. In brief, the freshly harvested *D. catenatum* stems (100 g) were washed with tap water, cut into small pieces, added with 500 mL of distiller water, and pulped twice with a household food processer. After centrifugation (2,683 *g*, 20 min), the supernatant was mixed with 20% (w/w) (NH_4_)_2_SO_4_, followed by 1.5 volumes of *t*-butanol, and then incubated at 25°C for 30 min under continuous stirring. After centrifugation at 2,683 *g* for 15 min, a clear three phases was formed. The upper phase containing *t*-butanol was recycled by vacuum evaporation. The lower (NH_4_)_2_SO_4_ phase was collected, precipitated by fourfold volumes of 95% ethanol overnight, centrifuged, dissolved, dialyzed (molecular weight cut-off: 3,500 Da), and lyophilized to obtain the DCP solid, which was sealed in an airtight plastic bag and stored at 4°C prior to testing. The steps for the extraction and separation of DCP from fresh *D. catenatum* stems *via* TPP and ethanol precipitation are graphically depicted in Figure 1.

### Structural Characterization of DCP

#### Chemical Component Analysis

The contents of carbohydrate, uronic acid, and soluble protein in DCP were measured through the assays of phenol–sulfuric acid, carbazole–sulfuric acid, and Bradford by using glucose (Glc), galacturonic acid (GalA), and BSA as standards, respectively (20–22).

#### Size Exclusion Chromatography Coupled With Multi-Angle Laser Light Scattering and Refractive Index (SEC-MALLS-RI) Analysis

The SEC-MALLS-RI analysis was used to determine the homogeneity, weight-average molecular weight (*M*_*w*_), number-average molecular weight (*M*_*n*_), molecular weight distribution (*M*_*w*_/*M*_*n*_), and *z*-average radius of gyration (*R*_*z*_) of the DCP. The HPSEC-MALLS-RI system was composed of a Waters 2695 HPLC (Waters, Milford, MA, USA), a MALLS detector (DAWN HELEOS II λ = 623.8 nm; Wyatt Technologies Corporation, USA), and a RI detector (Waters) equipped with a guard column (TSK PWXL, Japan) and a TSK G6000 PWXL column (7.8 mm × 30 cm, Japan) at 30°C. The eluant was 0.05 mol/L NaH_2_PO_4_ and 0.15 mol/L NaNO_3_ containing 0.02% (*w/w*) NaN_3_ (pH 7.0) at the flow rate of 0.5 mL/min. The refractive index increment (*dn/dc*) value was used as 0.146 mL/g (23).

#### Monosaccharide Composition Analysis

Following the method of Yan et al. (24), the monosaccharide constituents of the DCP were determined by using an ion chromatography (IC, ICS5000, American Diane Company, CA, USA) equipped with a pulsed amperometric detector (Au electrode) and a Carbopac PA-20 column (150 mm × 3 mm) at 30°C. After hydrolysis with 2.0 mol/L TFA at 110°C for 6 h, the DCP sample was evaporated, diluted, filtered, and detected. The eluant and flow rate were 2.0 mmol/L NaOH solution and 0.45 mL/min, respectively. Glc, mannose (Man), galactose (Gal), rhamnose (Rha), arabinose (Ara), xylose (Xyl), fucose (Fuc), fructose (Fru), glucosamine (GlcN), glucuronic acid (GlcA), and GalA were used as monosaccharide standards and detected in accordance with the procedures described above.

#### Fourier-Transform Infrared (FT-IR) Spectroscopy

The lyophilized DCP (1.0 mg) was mixed with KBr powder at a mass ratio of 1:100 (*w/w*) and pressed into a 1 mm transparent flake. The FT-IR spectrum of DCP was then obtained with a FT-IR spectrometer (Nexus 670, Thermo Nicolet Co., USA) varying in the range of 400–4,000 cm^−1^ with a resolution of 4 cm^−1^.

#### Nuclear Magnetic Resonance (NMR) Spectroscopy

The lyophilized DCP (30 mg) was dissolved in 0.5 mL of D_2_O to record its 1D (^1^H, and ^13^C) and 2D [heteronuclear single-quantum correlation spectroscopy (HSQC), and heteronuclear multiple-bond correlation spectroscopy (HMBC)] NMR spectra. The NMR spectra were recorded on a Bruker AVIII-600 NMR instrument (Bruker, Rheinstetten, Germany) at 25°C with tetramethylsilane as an external standard (δ 0.00 ppm).

### Rheological Analysis of DCP

The rheological behaviors of DCP at various concentrations (20–60 mg/mL) were carried out on a DHR-1 rheogoniometer (TA instruments, New Castle, USA) at 25°C according to the method of Cai et al. (25). A cone and plane geometry system of 40 mm cone diameter, 1° angle, and 29 μm gap was used. The steady shear behavior of the DCP was detected at shear rates ranging from 0.1 to 1,000 s^−1^. The dynamic viscoelastic behavior of the DCP was investigated by frequency sweep changing from 0.1 to 100 rad/s at a strain of 2% within the linear viscoelastic region. Also, storage modulus (*G*′) and loss modulus (*G*″) were obtained. Data collection and analysis were performed *via* the on-line Trios 3.0 software.

### *In vitro* Immunostimulatory Activity of DCP

#### Cell Culture

RAW264.7 murine macrophages cell line was supplied by American Type Culture Collection (Manassas, VA, USA). The cells were cultured in a dish containing DMEM supplemented with 10% FBS, 100 U/mL penicillin, and 100 μg/mL streptomycin in a humidified atmosphere of 5% CO_2_ at 37°C.

#### Cell VIAbility

Cell *via*bility was inspected using the CCK-8 assay. RAW264.7 cells were seeded in 96-well plates at a density of 5 × 10^4^ cells per well. After 24 h, the cells were incubated with DCP at different concentrations (25–200 μg/mL) for 24 h. After incubation, each well was added with 10 μL of CCK-8 reagent and incubated for 4 h. Absorbance at 450 nm was recorded on a microplate reader (Biotek, Winooski, VT, USA).

#### Assay for Macrophage Phagocytosis

RAW264.7 cells were seeded in a 96-well plate, incubated at 37°C in a humidified atmosphere of 5% CO_2_ overnight, and treated with various concentrations (25–200 μg/mL) of DCP and LPS (4 ng/mL) for 24 h. Afterward, each well was added with 100 μL of FITC-labeled *E. coli* and incubated at 37°C for 2 h. The cells were rinsed twice with PBS to wash away the FITC-labeled bacteria that were not phagocytosed by macrophages, then fixed with 4% paraformaldehyde, and finally photographed through an inverted microscope (Nikon Corporation, Tokyo, Japan).

#### Nitrite Assay

The amount of nitrite, an indicator of NO, was commonly detected by Griess reagent as an indicator of immunoenhancing activity. RAW264.7 cells were plated in 96-well cell culture plates with a density of 5 × 10^5^ cells/well. After 24 h, the cells were treated with DCP (25–200 μg/mL) and LPS (4 ng/mL) for another 24 h. Nitrite amount was measured using Griess reagent (1% sulfanilamide and 0.1% naphthylethylenediamine dihydrochloride in 2.5% phosphoric acid). Each well was added with 100 μL of cell culture medium mixed with 100 μL of Griess reagent and incubated for 30 min. Absorbance at 540 nm was measured.

#### Measurement of Cytokine Expression

RAW264.7 cells were seeded into a 96-well microplate at 5 × 10^5^ cells/well and incubated with or without DCP with the final concentration of 25–200 μg/mL. After incubation for 24 h at 37°C in a humidified atmosphere with 5% CO_2_, the culture supernatants were harvested for the measurement of cytokine contents (IL-1β, IL-6, and IFN-γ) by ELISA kits in accordance with the manufacturer's instructions. Absorbance at 450 nm was detected.

#### Statistical Analysis

All treatments in immunostimulatory activity assay were conducted in triplicate. GraphPad Prism 8 software was used for statistical analysis, and all data were presented as mean ± SEM. One-way ANOVA and Turkey's multiple comparison was used to analyze differences among groups. A level of *p* < 0.05 signified statistical significance and indicated as follows: ^*^p < 0.05, ^**^p < 0.01, ^***^p < 0.001.

## Results and Discussions

### Physicochemical Properties and Chain Conformation of DCP

**Figure 2:**
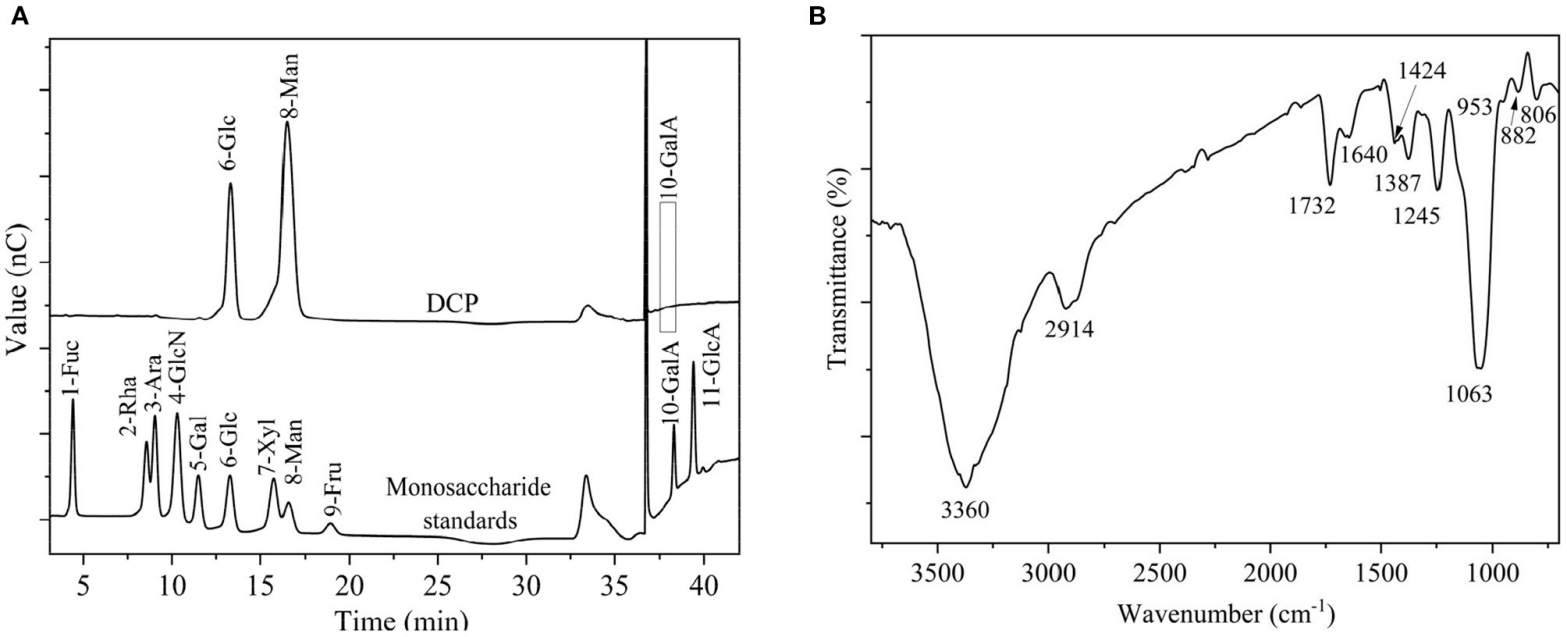
**(A)** Monosaccharide compositions of DCP and monosaccharide standards, and **(B)** FT-IR spectrum of DCP.

After TPP and ethanol precipitation, a water-soluble DCP was obtained from fresh *D. catenatum* stems with a yield of 15.2% ± 0.6% (Figure 1). The chemical composition, molecular parameters, and monosaccharide constituents of the DCP are summarized in Table 1. The contents of total carbohydrate and uronic acid in DCP were 92.75% ± 1.78% and 5.80 ± 0.05%, respectively, suggesting that this polysaccharide is acidic with high purity. According to the Bradford method, no protein was detected in the DCP. This finding indicated that TPP combined with ethanol precipitation could effectively remove free proteins in the DCP, and this finding was similar to our earlier research (26). Moreover, no characteristic absorption peaks at ~260 and 280 nm were found in the UV-vis spectrum of DCP (data not shown), thus further implying the absence of nucleic acids and protein. As illustrated in Table 1 and Figure 2A, the DCP was consisted of Glc and Man with a trace of GalA at a molar ratio of 30.2:69.5:0.3 according to monosaccharide standards. Therefore, Man and Glc were the predominant monosaccharides in the DCP. This outcome revealed that the DCP obtained from fresh *D. catenatum* stems with TPP and ethanol precipitation was an acidic glucomannan, which was inconsistent with the results reported by He et al. (27), Kuang et al. (28), and Zhang et al. (10). Liang et al. (29) found that the two polysaccharides (DOP1-DES and DOP2-DES) obtained from *D. officinale* stem *via* deep eutectic solvents were both mannoglucans comprising by Glc and Man in the percentages of 2.2:1.0 and 3.7:1.0, respectively. This finding was different from the above-mentioned data. Gal and Ara were also found in other reported *D. officinale* polysaccharides (30, 31). These differences in monosaccharide composition were mainly attributed the variation in the sources of *D. catenatum* plant and the preparation protocols.

**Table 1:** Chemical composition, molecular parameters, and monosaccharide constituents of the DCP isolated from fresh *D. catenatum* stems by TPP and ethanol precipitation.

**Sample**	**Yield**	**Sugar**	**Uronic acid**	**Protein**	***M_***w***_***	***M_***n***_***	***M_***w***_*/*M_***n***_***	***R_***z***_***	**Sugar composition (mol%)**		
	**(%)**	**(wt%)**	**(wt%)**	**(wt%)**	**(×10^**5**^ Da)**	**(×10^**5**^Da)**		***(nm)***			
									**Glc**	**Man**	**GalA**
DCP	15.2 ± 0.6	92.75 ± 1.78	5.80 ± 0.05	n.d.	2.21	2.18	1.01	17.9	30.2	69.5	0.3

*n.d. not determined*.

**Figure 3:**
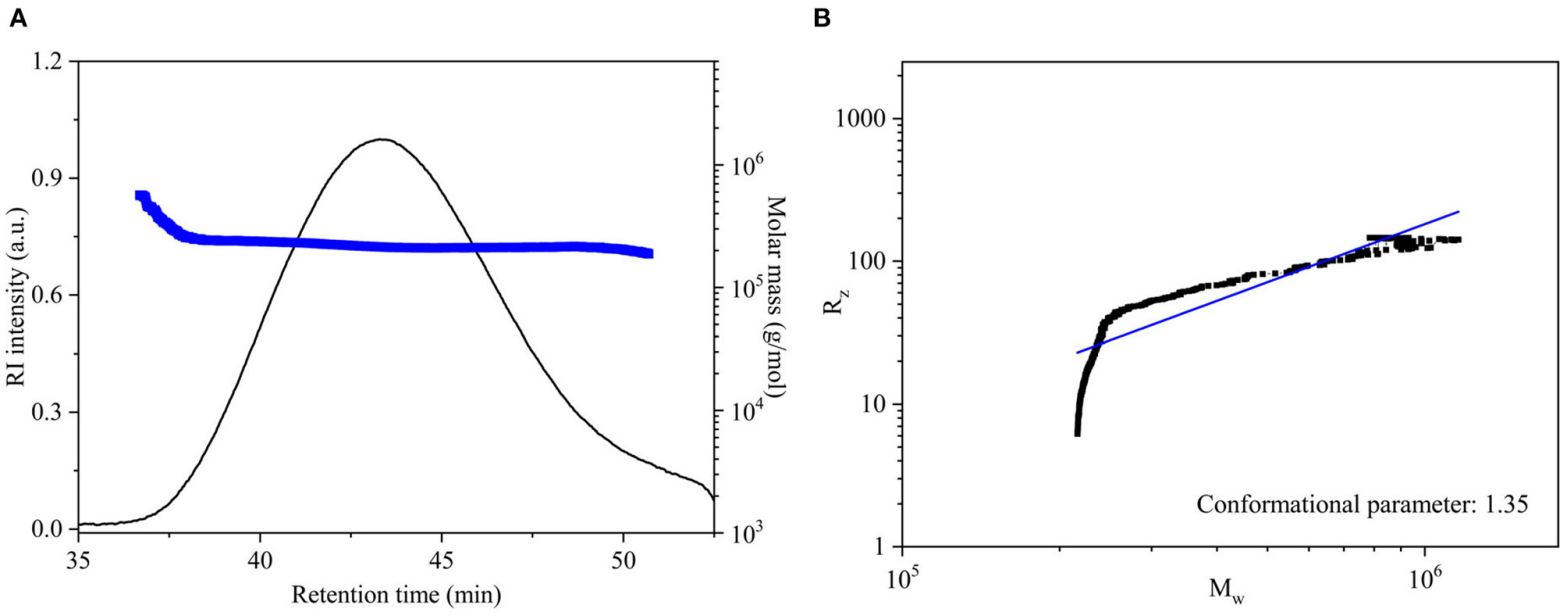
**(A)** SEC elution and molar mass profiles, and **(B)** logarithmic plot of *M*_*w*_ vs. *R*_*z*_ for DCP.

Figure 3A shows that the chromatographic peak of the DCP had a single symmetrical peak, which demonstrated that the DCP obtained from fresh *D. catenatum* stems through TPP and ethanol precipitation had high homogeneity within the measured molar mass range. Table 1 shows that the *M*_*w*_, *M*_*n*_, and *M*_*w*_/*M*_*n*_ of the DCP were 2.21 × 10^5^ Da, 2.18 × 10^5^ Da, and 1.01, respectively. The *M*_*w*_ of DCP was larger than those of previously reported polysaccharides extracted from dried *D. officinale* stems (6.8 × 10^3^-1.78 × 10^5^ Da) (8, 28, 31–33). The DCP with high *M*_*w*_ would be beneficial for an enhanced immunostimulatory activity, which was verified in Section *in vitro* Immunostimulatory Activity of DCP. In addition, its *M*_*w*_/*M*_*n*_ (1.01) value further indicated that the DCP was a highly homogenous polysaccharide.

The chain conformation of polysaccharide in solution can be estimated by the relationship between *M*_*w*_ and *R*_*z*_ (*R*_*z*_ = *k*·$M_{w}^{\alpha }$) obtained from the SEC-MALLS-RI analysis (18). In general, the conformational parameter α of 0.3, 0.5–0.6, and ≥1.0 corresponds to the chain conformation of spheres, flexible chains, and rigid rods, respectively. In this work, the logarithmic plot describing the relationship between the *M*_*w*_ and *R*_*z*_ (*R*_*z*_ = *k*·$M_{w}^{\text{α}}$) of DCP is displayed in Figure 3B. The value of α for DCP was 1.35, which was higher than 1.0, revealing that the DCP presented as an extended rigid rod in aqueous solution (pH 7.0).

### Structural Features of DCP

Figure 2B displays the FT-IR spectrum of DCP. The DCP exhibited typical characteristic bands of polysaccharides. The wide and strong stretching vibration band at ~3,360 cm^−1^ signified the existence of O-H group, and the small and feeble stretching vibration at ~2,914 cm^−1^ corresponded to the C-H group of methyl and methylene groups. The signals at ~1,732 and 1,640 cm^−1^ corresponding to stretching vibrations of C=O of O-acetyl (-O-COCH_3_) and carboxylic (COO-) groups, respectively (34). Therefore, the DCP might be an acidic or *O*-acetylated glucomannan, and this finding was in accord with the findings of Table 1 and previous studies (32, 33, 35). The peak near 1,424 cm^−1^ was associated with stretching vibration of the C-O-H group. Signals near 1,387, 1,245, and 1,063 cm^−1^ were separately related to bending vibration of C-H group, and stretching vibrations of C–O and C–O–C groups. The characteristic absorption peaks at ~953, 886, and 808 cm^−1^ indicated that the DCP had the α- and β-configurations of Man and Glc in pyranose sugar forms, respectively (29).

**Figure 4:**
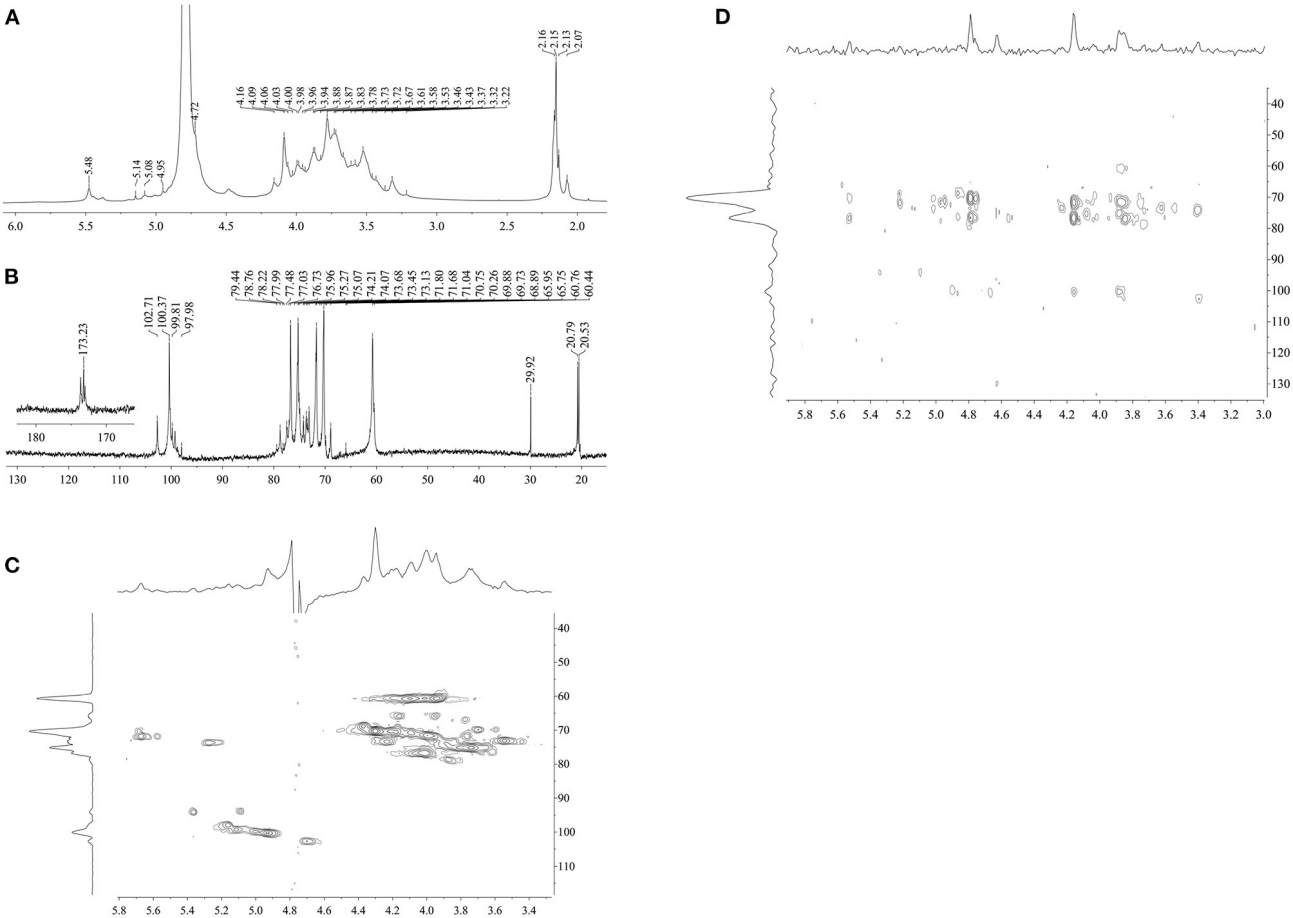
**(A)** ^1^H NMR, **(B)** ^13^C NMR, **(C)** HSQC, and **(D)** HMBC of DCP.

The structural feature of DCP was further characterized by using 1D (^1^H, and ^13^C) and 2D (HSQC and HMBC) NMR measurement, and the obtained results are illustrated in Figure 4 and Table 2. In the ^1^H NMR (Figure 4A) and ^13^C NMR (Figure 4B) spectra, the signals of anomeric protons (H-1) and carbons (C-1) were primarily within the ranges of δ 4.69–5.48 ppm and δ 97.98–102.69 ppm, respectively. Among which, the strong signal at δ 4.79 ppm was attributed to the HDO of D_2_O. This result indicated that both α- and β-configurations are present in the DCP. As shown in Figure 4A, the signals at δ 2.07–2.16 ppm in the ^1^H NMR spectrum corresponded to the –CH_3_ group of *O*-acetyl groups. In the ^13^C NMR spectrum of DCP (Figure 4B), the signals at δ 173.23 ppm and δ 20.79 (δ 20.53) ppm were separately ascribed to the –COOH and –CH_3_ groups of *O*-acetyl groups. These findings suggested the existence of O-acetyl groups in the DCP, which was in line with the FT-IR analysis (Figure 2B) and previously reported data (33, 35). The presence of *O*-acetyl groups in DCP might be responsible for the enhanced phagocytic activity of RAW264.7 cells during immunoprotection (34), which was further confirmed in Section *In vitro* Immunostimulatory Activity of DCP.

**Table 2:** ^1^H and ^13^C NMR chemical shift (δ, ppm) assignments for DCP.

**Sugar residues**	**Chemical shifts (** ***δ*** **, ppm)**					
	**H-1/C-1**	**H-2/C-2**	**H-3/C-3**	**H-4/C-4**	**H-5/C-5**	**H-6/C-6**
(1→4)-β-D-Manp	4.72/102.71	4.09/71.04	3.87/74.21	3.83/76.73	3.61/77.03	3.94(3.73)/60.44
2-*O*-acetyl-(1→4)-β-D-Manp	4.95/100.37	5.48/71.68	4.06/70.75	3.88/75.96	3.67/75.27	4.00(3.73)/60.44
(1→6)-α-D-Glcp	5.08/99.81	3.53/73.68	3.72/73.68	3.43/73.45	3.94/74.07	3.87(3.78)/65.95
(1→4)-α-D-Glcp	5.14/97.98	3.58/73.13	3.72/69.88	3.67/75.07	4.16/65.75	3.83(3.78)/60.76

The anomeric signals at δ 4.72, 4.95, 5.08, and 5.14 ppm in the ^1^H NMR spectrum (Figure 4A) and δ 102.71, 100.37, 99.81, and 97.98 ppm in the ^13^C NMR spectrum (Figure 4B) corresponded to the H-1/C-1 of (1→4)-β-D-mannopyranosyl (Manp), 2-*O*-acetyl-(1→4)-β-D-Manp, (1→6)-α-D-glucopyranosyl (Glcp), and (1→4)-α-D-Glcp of DCP, respectively (8, 31, 36). As displayed in Figure 4A, the signal at δ 5.48 ppm belonged to the H-2 of 2-*O*-acetyl-(1→4)-β-D-Manp. This outcome showed that the DCP comprised four types of sugar residues, which were further identified by HSQC (Figure 4C). No signals were observed at δ 82–88 ppm (feature of furanoses) in the ^13^C NMR spectrum (Figure 4B), implying that the DCP was dominantly consisted of pyranose sugars (34). All assignments of other proton (H-2 to H-6) and carbon (C-2 to C-6) signals for the four sugar residues were confirmed and are listed in Table 2 according to HSQC (Figure 4C) and HMBC (Figure 4D) spectra coupled with published data on *D. officinale* polysaccharides (6, 8, 28, 31, 33, 35). On the basis of these results, the DCP derived from fresh *D. catenatum* stems *via* TPP and ethanol precipitation was classified as glucomannan and mainly composed of (1→4)-β-D-Manp, 2-*O*-acetyl-(1→4)-β-D-Manp, (1→6)-α-D-Glcp, and (1→4)-α-D-Glcp residues, with Man and Glc as the predominant components.

### Rheological Properties of DCP

**Figure 5:**
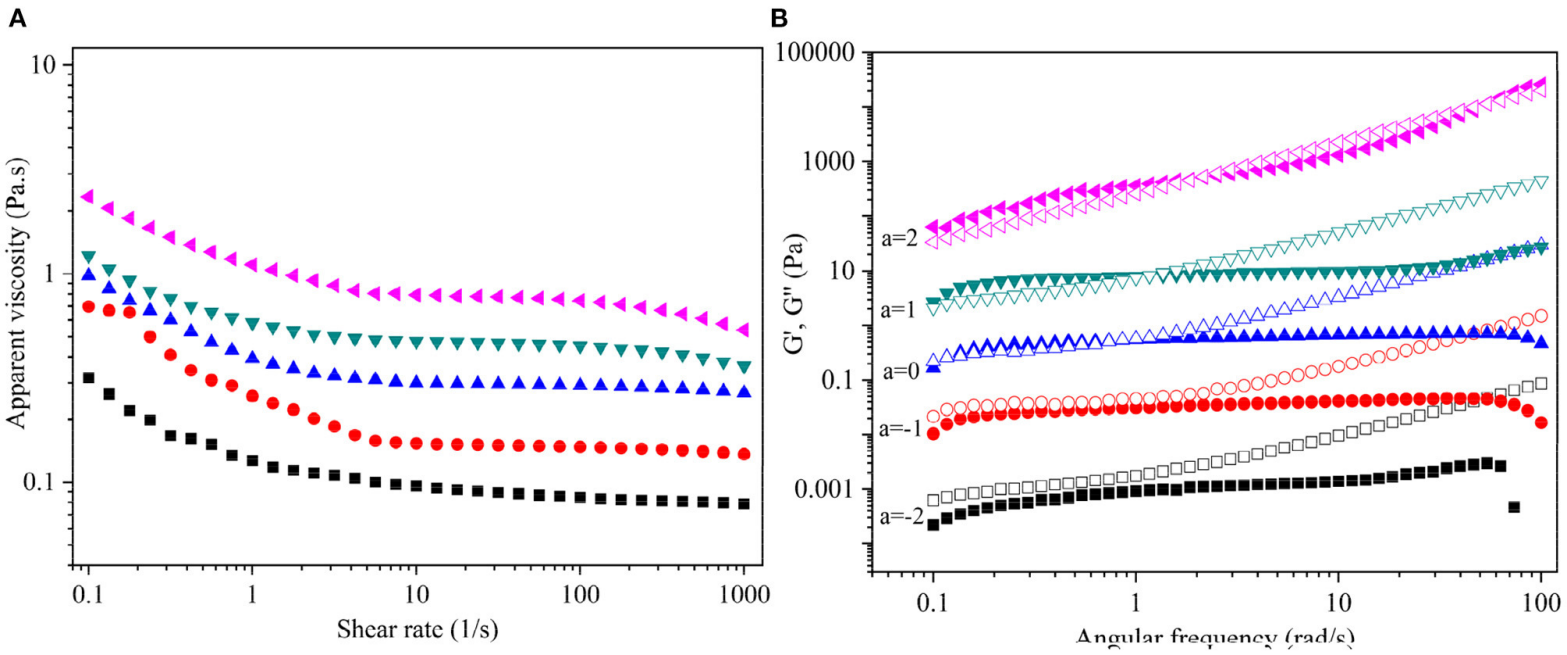
**(A)** Dependence of apparent viscosity (η_*a*_) on shear rate (γ), and **(B)** Plots of storage modulus *G*′ (solid symbols) and loss modulus *G*^′′^ (open symbols) on angular frequency (ω) for DCP solution at different concentrations and 25°C. The DCP concentrations are 20, 30, 40, 50, and 60 mg/mL from bottom to top. The data were shifted along vertical axis by 10^*a*^ to avoid overlap.

Figure 5A illustrates the steady-state fluid behavior of aqueous DCP at 20–60 mg/mL concentrations and 25°C. It was noted that the apparent viscosity (η_*a*_) of DCP solutions was positively correlated with their concentrations but negatively correlated with the shear rate (γ). Figure 5A shows that the η_*a*_ of DCP decreased with the increase in γ within the measured concentration range (20–60 mg/mL), suggesting that the DCP exhibited a non-Newtonian shear-thinning behavior in aqueous solution. This outcome might be due to the breakage of the entwined polysaccharide chains by shear force (25). Moreover, the shear-thinning nature of DCP was highly evident at low γ (0.1-10 s^−1^). An analogous phenomenon was also reported in okra polysaccharides (18, 37) and yeast mannans (38). At a high γ (>10 s^−1^), a near-Newtonian plateau was observed in DCP solutions, thus providing the ability to re-establish the entanglements of the disrupted polysaccharide chains. The shear-thinning character of the DCP solution was also quantitatively assessed through the Power-law model (σ = *k*·γ^*n*^), where σ, *k*, and *n* are the shear stress (Pa), consistency coefficient (Pa.s), and flow behavior index, respectively. In general, the *n* values of 0–1.0, 1.0, and >1.0 reflect shear-thinning, near-Newtonian fluid, and shear-thickening behaviors, respectively (25). Supplementary Table S1 shows that the determination coefficients (*R*^2^) were all >0.99, which suggested that the Power-law model was reliable for describing the shear-thinning behavior of DCP in aqueous solution. The value of *n* decreased from 0.971 to 0.820, whereas that of *k* increased from 0.096 to 1.759 Pa.s as the DCP concentration increasing from 20 to 60 mg/mL. In agreement with Figure 5A, these results implied that the DCP solution exhibited pronounced shear-thinning nature with high viscosity at high concentrations.

The dynamic viscoelastic behavior of DCP solution at different concentrations (20–60 mg/mL) and constant strain levels of 2% and 25°C is shown in Figure 5B. The *G*′ and *G*″ of the DCP exhibited concentration- and frequency-dependent behavior across the tested angular frequency (ω) range from 0.1 to 100 rad/s. This outcome was in consistent with the before report of Cai et al. (25). At concentrations ≤30 mg/mL, the *G*″ was always greater than the *G*′ throughout the entire range of ω (Figure 5B), indicating that the DCP predominantly exhibited a fluid-like behavior. At a low ω, the *G*′ was almost equal to *G*^′′^ with the increasing concentration. At 40 mg/mL, an intercept of *G*′ and *G*^′′^ was found at low ω, suggesting that the DCP presented viscoelastic property. When the ω was less than the intercept, that is, *G*′ > *G*^′′^, the DCP solution showed a solid-like nature. When ω exceeded the intercept, that is, *G*′ < *G*^′′^, the DCP solution exhibited a liquid-like nature. Moreover, the intercept of *G*′ and *G*^′′^ gradually moved to a high ω as the concentration further increased from 40 to 60 mg/mL (Figure 5B). This finding indicated that the viscoelastic behavior of DCP solution became evident.

### *In vitro* Immunostimulatory Activity of DCP

**Figure 6:**
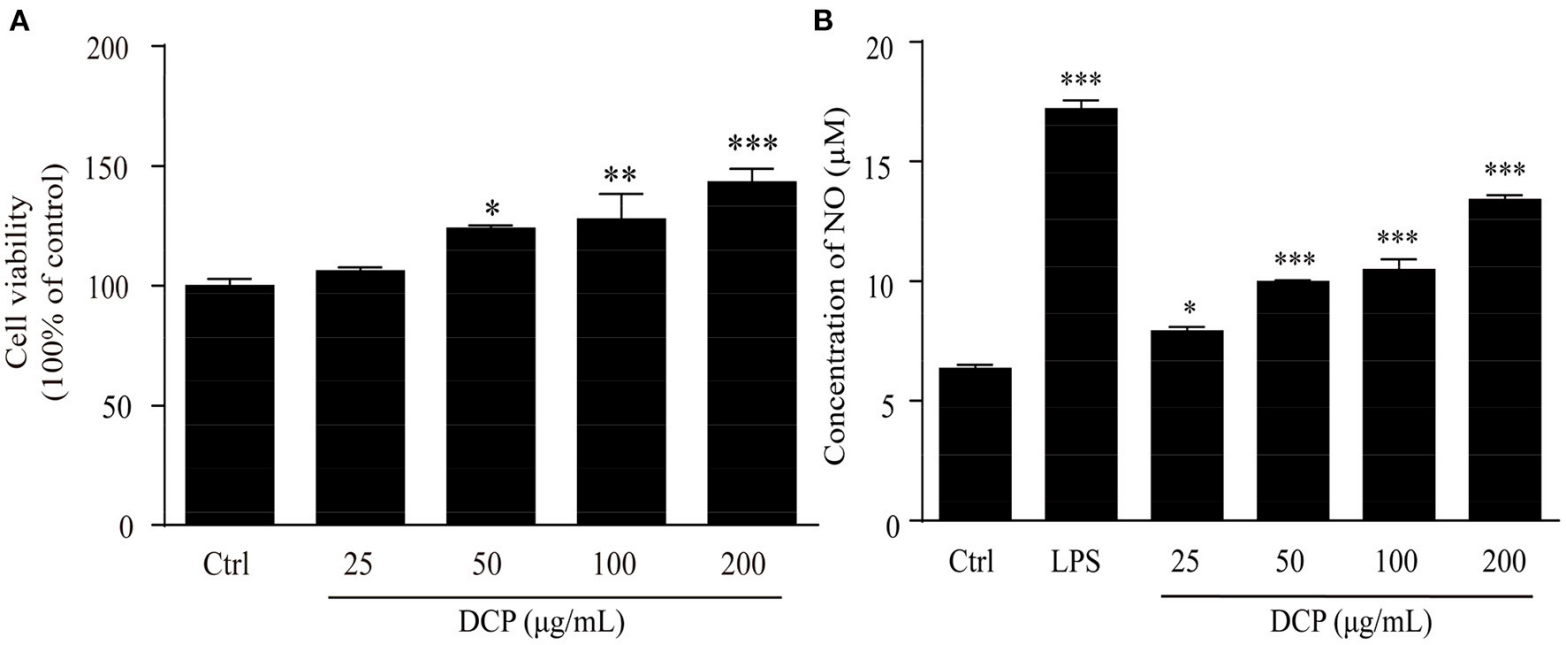
Effect of DCP at different concentrations on **(A)** the proliferation of RAW264.7 cells, and **(B)** NO production in RAW264.7 cells (*n* = 3, ± SEM). ^*^p < 0.05, ^**^p < 0.01, ^***^p < 0.001 vs. the control or LPS group.

Macrophages are one of the most significant immune cells of the innate immune system and play a substantial role in the body's defense against viral infections (39). These cells produce inflammatory mediators to remove pathogens and repair tissue damage, thereby responding to microbial antigens (40). CCK-8 assay was performed after DCP addition to investigate the effects of DCP on cell *via*bilities and determine the best concentrations for further studies. As depicted in Figure 6A, the DCP could promote macrophage proliferation. This finding revealed that different concentrations of DCP extracted from fresh *D. catenatum* stems did not inhibit the growth of RAW264.7 cells within the measured concentration range (25–200 μg/mL). Conversely, cell proliferation was induced when the DCP concentration varying from 50 to 200 μg/mL (*p* < 0.05), indicating that the DCP can promote macrophage proliferation. At the same concentration, the ability of DCP to promote cell proliferation was remarkably higher than that reported in literature (34). This finding showed that the DCP with high *M*_*w*_ exhibited good immunostimulatory activity. Compared with control group, the DCP obtained from fresh *D. catenatum* stems (25–200 μg/mL) significantly and dose-dependently upregulated the NO production (*p* < 0.05) (Figure 6B). When the RAW264.7 cells were treated with 200 μg/mL DCP, the NO production reached 13.44 ± 0.50 μM, which was higher than those under the treatment of *D. officinale* polysaccharide fractions (DOP-1 and DOP-2) reported by Xia et al. (30). These results suggested that DCP could stimulate macrophages to produce inflammatory mediators (e.g., NO) and activate the immune system. Moreover, the tendency of DCP to promote NO secretion was in accord with the outcome of macrophage proliferation (Figure 6A).

**Figure 7:**
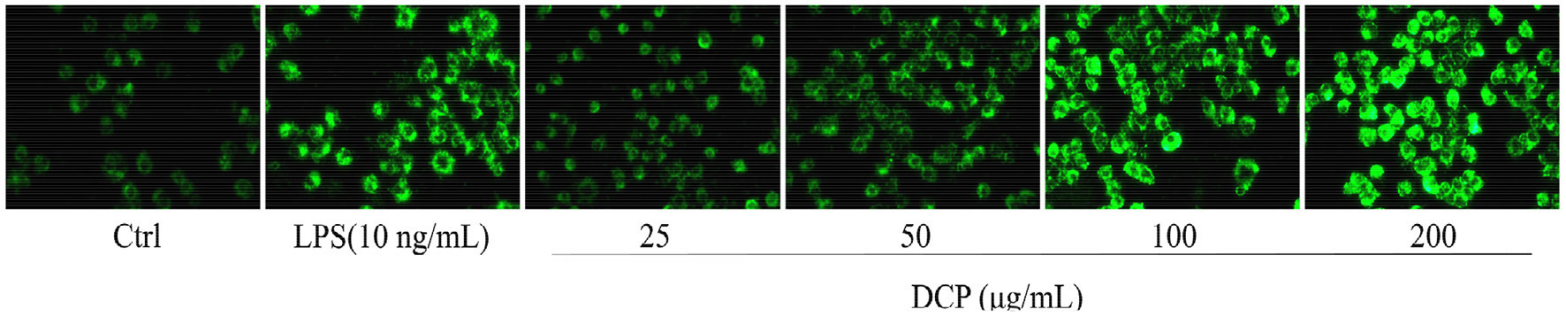
A fluorescence microscope image of RAW264.7 cells stained with FITC-labeled *E. coli* (×400).

Figure 7 displays the immune enhancement effect of DCP on macrophage phagocytosis measured by internalized FITC-labeled *E. coli* particles by using a fluorescence microscope, which can provide visual evidence of phagocytic uptake. One of the main tasks of phagocytes is to detect, engulf, and digest foreign objects and apoptotic cell debris by approaching the prey, thus effectively devouring the prey at the highest possible rate (41). Compared with the untreated RAW264.7 macrophages, the cells treated with DCP or LPS for 24 h showed increased fluorescence intensity after the absorption of FITC-labeled *E. coli*. The RAW264.7 cells treated with 100–200 μg/mL DCP had stronger fluorescence intensity than the LPS-treated cells, especially at 200 μg/mL. These data revealed that DCP improved the phagocytic ability and non-specific immunity of RAW264.7 cells within the tested concentration range. Therefore, this polysaccharide can effectively improve the recognition rate of macrophages. Moreover, the fluorescence intensity of RAW264.7 cells treated with DCP in this study was stronger than those of DOP fractions (DOPW-1 and DOPW-2) reported by Tao et al. (6). This finding revealed that the novel DCP exerted more pronounced phagocytic ability compared with DOPW-1 and DOPW-2 due to its higher content of *O*-acetyl groups. Therefore, the presence of *O*-acetyl groups may be responsible for the substantially enhanced phagocytic activity of RAW264.7 cells during immune protection.

**Figure 8:**
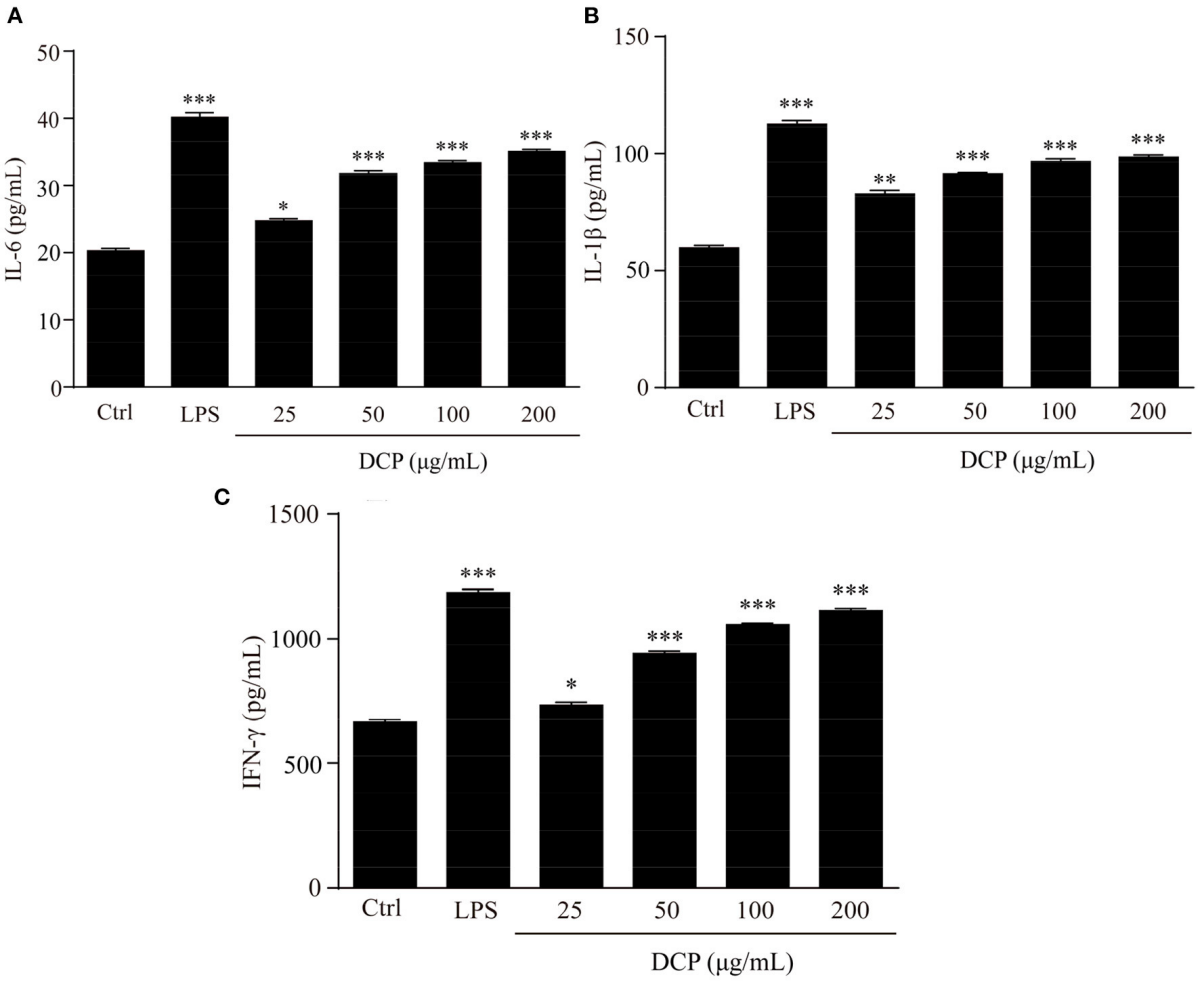
Effects of DCP at different concentrations on the levels of **(A)** IL-6, **(B)** IL-1β, and **(C)** IFN-γ in RAW264.7 cells. (*n* = 3, ±SEM). ^*^p < 0.05, ^**^p < 0.01, ^***^p < 0.001 vs. control group.

The effect of DCP on cytokine production is shown in Figure 8. As pro-inflammatory cytokines, IL-6, L-1β, and IFN-γ, are critical to the innate immune response of bacteria in cells (42, 43). Macrophages primed with IL-6, L-1β, and IFN-γ rapidly respond to a secondary signal, resulting in their tumoricidal and microbicidal activities that are essential for adaptive immunity against viral and infections (44, 45). In this study, the DCP dose-dependently and significantly enhanced the secretion of macrophage-related cytokines, such as IL-6, L-1β, and IFN-γ, in RAW264.7 macrophages within the determined concentration range of 50–200 μg/mL as compared to that in the control group cells (*p* < 0.05). At 200 μg/mL, the concentrations of IL-6, L-1β, and IFN-γ of DCP from RAW264.7 macrophages were 33.89 ± 0.69, 100.02 ± 2.12, and 1126.04 ± 18.98 pg/mL, respectively, which were close to those under LPS treatment. This outcome revealed that the DCP effectively stimulated the secretion of cytokines, which in turn activated the macrophages. Xia et al. (30) found that the IL-1β production rate in RAW264.7 cells treated with 200 μg/mL DOP-1 and DOP-2 was within 81.1 ± 0.8 and 90.0 ± 2.2 pg/mL, respectively, which was lower the present result for IL-1β secretion. Therefore, the DCP could help RAW264.7 release cytokines and enhance the body's immune activity.

## Conclusions

In summary, the present study demonstrated that a novel immunostimulating polysaccharide DCP was obtained from freshly harvested *D. catenatum* stems through TPP coupled with ethanol precipitation. The DCP was an acidic *O*-acetylated glucomannan and comprised by Glc, Man, and GalA in the molar ratio of 30.2:69.5:0.3 with an *M*_*w*_ of 2.21 × 10^5^ Da, and existed as an extended rigid rod in aqueous solution with good steady-state fluid and dynamic viscoelastic properties. DCP exhibited beneficial effects on immunostimulation in RAW264.7 cells by promoting macrophage proliferation and NO production, enhancing phagocytic activity and stimulating cytokines secretion. Therefore, the DCP obtained from fresh *D. catenatum* stems can be developed as a potential immunostimulator for functional food, medicine, and cosmetics uses.

## Data Availability Statement

The raw data supporting the conclusions of this article will be made available by the authors, without undue reservation.

## Author Contributions

JL, LY, JS, YZ, LZ, and JY: designed the experiments. LY, CW, JL, and HX: performed the experiments. JL, LY, and JY: analyzed the data and wrote the manuscript. JY and LZ: reviewed the manuscript. All authors contributed to the article and approved the submitted version.

## Funding

This work was supported financially by the National Key R&D Program of China (2017YFC1702201), the National Natural Science Foundation of China (31671812, 31970316, 32170274), Shanghai Academic Research Leader (19XD1405000), and Opening Foundation of the State Key Laboratory of Subtropical Silviculture, Zhejiang A&F University (Grant no. 2018FR003, ZY20180206).

## Conflict of Interest

The authors declare that the research was conducted in the absence of any commercial or financial relationships that could be construed as a potential conflict of interest.

## Publisher's Note

All claims expressed in this article are solely those of the authors and do not necessarily represent those of their affiliated organizations, or those of the publisher, the editors and the reviewers. Any product that may be evaluated in this article, or claim that may be made by its manufacturer, is not guaranteed or endorsed by the publisher.
