# Supplementary material for: Preparation, Structural Features and in vitro Immunostimulatory Activity of a Glucomannan From Fresh Dendrobium catenatum Stems
Source: Front Nutr. 2022 Feb 1;8:823803. doi: 10.3389/fnut.2021.823803 (PMC8843939; doi:10.3389/fnut.2021.823803)
Supplement: Supplementary file 1 [file Table_1.doc]

**Supplementary Data:**

**Table S1. Steady state flow parameters of DCP at different concentrations as calculated based on Power-law model.**

| DCP concentration (mg/mL) | *k* (Pa.s) | *n* | R^2^ |
| --- | --- | --- | --- |
| 20 | 0.096 | 0.971 | 0.999 |
| 30 | 0.187 | 0.952 | 0.999 |
| 40 | 0.366 | 0.934 | 0.998 |
| 50 | 0.847 | 0.870 | 0.996 |
| 60 | 1.759 | 0.820 | 0.999 |
